# Brain Implants to Erase Memories

**DOI:** 10.3389/fnins.2017.00584

**Published:** 2017-10-24

**Authors:** Walter Glannon

**Affiliations:** Philosophy/Arts, University of Calgary, Calgary, AB, Canada

**Keywords:** disorders of memory content, electrical brain stimulation, fear memory, memory erasure, memory trace, reconsolidation blockade

Implantable devices in the brain can reestablish functional connectivity in neural circuits disrupted in major depression, obsessive-compulsive disorder, and other psychiatric disorders. The most frequently used device of this type is deep brain stimulation (DBS) (Benabid, 2007; Lozano and Lipsman, 2013) Other neural implants can restore or improve certain neural functions lost or impaired through traumatic brain injury, infection, or other insults to the brain. One example is a hippocampal prosthesis (Berger et al., 2011; Hampson et al., 2013). This is a complex array of electrodes implanted in a region involving connections between the hippocampus and the entorhinal cortex. Although it is still at the experimental stage, a hippocampal prosthesis could restore the ability to encode new memories for people with damage in this brain region.

Instead of enabling the formation of new memories, could a device implanted in the brain erase memories that have been encoded, consolidated, and reconsolidated? DBS can modulate dysfunctional circuits mediating sensorimotor, cognitive, and emotional processing. Theoretically, this or a similar stimulating technique could selectively erase a pathological fear memory by inactivating neurons and excitatory synapses constituting the memory trace. This could disrupt reconsolidation of the memory stored as information in the brain. Erasing fear memories identified as the source of anxiety, panic, phobia, and post-traumatic stress disorder (PTSD) could be an effective therapy when they fail to respond to other treatments (Pitman, 2015).

Neuroscientists can use PET or fMRI to measure changes in neural activity and synaptic connectivity following manipulation of neural circuits associated with different memory systems. Neuroimaging could confirm erasure of a memory trace based on these changes. Hypothetically, electrical stimulation from an implantable device like DBS could reduce activity in neurons constituting the emotionally charged memory trace underlying conditioned responses to aversive stimuli and enable a subject to unlearn pathological behavior. In the psychiatric disorders I have mentioned, the emotional representation, or trace, of a memory of a disturbing or traumatizing event remains embedded in the brain beyond any short-term adaptive function. This disrupts the memory network regulating fear and results in pathological thought and behavior (Parsons and Ressler, 2013). Anxiety, panic, phobia, and PTSD are disorders of memory *content* associated with the emotional representation of the memory. These are distinct from different forms of amnesia, which is a disorder of memory *capacity* (Kopelman, 2002). The problem is not an inability to form memories, but an inability to extinguish them.

The source of these disorders is hyperactivity in the basolateral amygdala of the fear memory network. This occurs when a negative emotional memory of a fearful experience or a series of such experiences forms and solidifies in the brain through the processes of consolidation and reconsolidation. One theory of fear memory consolidation following a traumatic experience is that the memory embeds in the amygdala from the release of noradrenaline in response to the subject's stress reaction to the experience. The memory becomes more firmly embedded in this brain region from behavior in which the subject learns to link an aversive stimulus with a conditioned stimulus. Memories must be updated constantly to remain stored in the brain. Updating memories consists in reconsolidating them after retrieval. This process serves an adaptive purpose by enabling the subject to make information in the brain relevant to current and future circumstances (Nader et al., 2000; Nader and Einarsson, 2010). One goal of memory research is to interfere with reconsolidation during or immediately after retrieval in order to weaken or erase a traumatic memory trace. Memories are labile and susceptible to alteration at this time. Psychiatrist Roger Pitman explains how this process would occur: “For reconsolidation blockade, or updating, to be successful, two steps are required. First, the problematic memory must be destabilized. Second, its re-stabilization (reconsolidation) must then be prevented or modified (updated)” (Pitman, 2011, p. 2, 2015). Pharmacological intervention is one way of disrupting reconsolidation. In several studies, the beta-adrenergic receptor antagonist propranolol attenuated the emotionally charged content of a traumatic memory in a number of subjects (Pitman et al., 2002; Kindt et al., 2009; Soeter and Kindt, 2015). Yet if the drug weakens but does not erase the emotional representation of the memory, certain stimuli could reactivate its emotionally charged content. This could reintroduce the psychopathology.

A different and potentially more effective way of blocking reconsolidation would be to eradicate the emotional representation of the memory. In principle, certain drugs could block reconsolidation of the fear memory by blocking protein synthesis in the basolateral amygdala where the memory trace was located. During retrieval, infusion of a protein synthesis inhibitor such as anisomycin in this brain region might disrupt reconsolidation and effectively erase the memory trace (Schacter and Loftus, 2013). This or a similar acting drug would also interfere with long-term potentiation (LTP) and the transcription factor cyclic-response element-binding protein (CREB) that regulate protein synthesis in the formation and retention of memories. If they were effective, these processes would rule out the possibility of repeating a heightened emotional reaction to stimuli associated with the memory trace because there would no longer be any trace in the brain (Agren et al., 2012).

A major challenge to pharmacological erasure of memory is the selectivity of this intervention. Many memories of fearful experiences are adaptive and critical to survival because they enable us to recognize and react appropriately to threatening situations. Not all fear memories are pathological or maladaptive. Because of the distributed and non-discriminating effects of psychotropic drugs, a drug intended to erase the trace could have unintended expanding effects and impair normal functions of the fear memory system. A drug infused in the brain could alter both targeted and non-targeted nuclei in the limbic system and alter normal emotional processing. This could introduce a new psychopathology.

The more focused action of deep brain electrical stimulation of the neurons and synapses within the memory trace might be able to overcome the problem of selectivity. Direct stimulation of these constituents of the trace at the critical frequency could neutralize the effects of LTP, CREB, and protein synthesis on the persistence of the memory. It could remove any obstacles to destabilizing and removing the memory as stored information in the brain. In addition, by precisely targeting the neurons within the trace, electrical stimulation could reduce the risk of expanding effects on adaptive fear memories and positive emotional memories. Combined with its neuromodulating action, the ability of DBS to probe circuits and nodes within these circuits would enable investigators to monitor its effects on the critical neurons and synapses (Lozano and Lipsman, 2013). This could prevent adverse expanding effects on neurons and synapses unrelated to the problematic memory. Lower-frequency deep brain electrical stimulation may be limited in only attenuating the emotional content of the memory trace. The outcome of the stimulation would be similar to that of propranolol because it would not eliminate this content. Higher-frequency stimulation could selectively inactivate neurons and synapses in the basolateral amygdala identified as the loci of the pathological fear memory trace, disrupt reconsolidation of the memory and erase it. Unlike thermoelectric and radiofrequency neural ablation, electrical stimulation could erase the trace without destroying brain tissue.

The selectivity problem is a problem about localization. The main question is whether a particular maladaptive fear memory would be localized enough for DBS to erase it while leaving adaptive fear memories intact. One hypothesis that could support the idea of selectively erasing a maladaptive fear memory is that functional imaging could reveal higher levels of activation in the nuclei associated with that memory when a subject was asked to recall the traumatic experience as the source of it (Pitman et al., 2002; Pitman, 2015). DBS could target these nuclei for inactivation and erasure.

Even if imaging showed localized metabolic overactivation in nuclei associated with the memory, there are questions about whether DBS could inactivate it. Although many studies have confirmed the neuromodulating effects of DBS, the mechanisms of action of the technique are not well understood. DBS increased glucose metabolism in the entorhinal cortex of a group of epilepsy patients and enhanced learning and spatial memory (Sulthana et al., 2012; Fell et al., 2013). Similarly, increasing metabolism in the fornix with DBS may be a way of enhancing memory in mild Alzheimer's disease (Lozano et al., 2016). The goal of using DBS to *enhance* certain types of memory in these studies is in contrast to the goal of using DBS to *erase* other types of memory. Enhancing memory would require activating metabolically underactive nuclei associated with LTP, CREB, and protein synthesis. Erasing memory would require inhibiting metabolically overactive nuclei associated with these same processes. The second mechanism would be similar, in some respects, to the modulating effects of high-frequency DBS on metabolically overactive circuits in treatment-resistant depression (Mayberg et al., 2005). One significant difference between DBS for depression and DBS for memory erasure, however, is that the target in the second case would be more discrete. Whether DBS enhanced memory or erased it would depend on the frequency of the electrical current delivered to the targeted nuclei and its effect on the neurons constituting the memory trace.

Although direct stimulation of the hippocampus and medial temporal lobes can disrupt episodic memory (Merkow et al., 2017), it is unclear whether stimulation would have the same effects in inactivating a fear memory. Because it is hypothetical and involves many open neurophysiological questions, one must be appropriately circumspect in suggesting DBS as a technique of selective memory erasure. Yet with advances in its level of precision, it is in principle possible that this could be a novel application of DBS in the near future.

Suppose that investigators could use electrical stimulation from a device implanted in the brain to erase not just pathological fear memories but also less emotionally charged memories of disturbing experiences. If the emotional representation of some of these memories was localized in discrete limbic nuclei, the critical neurons, and excitatory synapses could be inactivated and the memory trace erased by DBS, should it be?

Remembering mistaken choices and actions can haunt one for years and make one indecisive when choosing between alternative courses of action in the present and future. Yet memories of these mistakes and of more emotionally disturbing experiences are necessary for character development and moral growth. They promote this development and growth by enabling the moral emotions of remorse and regret. They also enable us to reflect on our motivational states in forming and executing action plans that promote prudence and moral sensitivity toward others. Disturbing memories can provide constraints on decision-making that are necessary for effective rational and moral agency. Erasing a few disturbing memories might not undermine or impede the development and exercise of these capacities. However, erasing a broader set of memories could weaken agency and have deleterious effects on one's behavior. The mental capacity to integrate both positive and negative episodic memories in a unified autobiography also enables one to construct meaning from them. Deleting more than a critical number of memories could disrupt the psychological connectedness and continuity that constitute personal identity, the experience of persisting through time as the same person (Parfit, 1984; Tulving, 2002). Episodic and emotional memories are critical to agency and identity. It is not known whether or how removing some of these memories might affect these two fundamental aspects of human experience and cause harm.

Research into manipulating the content of memory has been limited to animal models. Psychiatric researchers will have to address many theoretical and technical challenges in moving from animal to first-in-human studies. Functional neuroimaging will be critical in identifying changes in brain activity correlating with a weakened or erased memory trace at neuronal and synaptic levels. The most important question is whether the critical neurons can be altered at a localized, discrete level. The idea of erasing a particular pathological fear memory with DBS or similar implantable electrical stimulation devices is still speculative. Yet it could become a way of treating what are currently treatment-resistant psychiatric disorders of memory content.

## Author contributions

The author confirms being the sole contributor of this work and approved it for publication.

### Conflict of interest statement

The author declares that the research was conducted in the absence of any commercial or financial relationships that could be construed as a potential conflict of interest.
